# Improving prediction accuracy of laser-induced shock wave velocity prediction using neural networks

**DOI:** 10.1038/s41598-024-63616-5

**Published:** 2024-06-12

**Authors:** Haoyu Yang

**Affiliations:** College of Computing, Georgia Institute of Technology, Atlanta, GA 30332 USA

**Keywords:** Shock wave velocity, Prediction, Neural networks, Laser parameters, Accuracy, Neuroscience, Mathematics and computing

## Abstract

The velocity of laser-induced shock waves affects the efficiency and efficacy of laser-based processes. The ability to accurately estimate shock wave velocity is critical for optimizing experimental combinations, creating laser-based systems, and assuring desired results. Traditional approaches to predict shock wave velocity involve empirical equations and analytical models based on simplified assumptions. However, these methods often lack accuracy and fail to capture the complex dynamics of laser-matter interactions. To overcome these limitations, we used a combination of an artificial neural network and a genetic algorithm to predict shock wave velocity. In this method, the neural network structure is dynamically designed. The optimization method does this by modifying the neural network's weights and figuring out the network's structure on our behalf. Based on the findings, our suggested technique worked very well; it surpassed other comparison methods by achieving the lowest average errors in terms of RMSE and MAE, which are 4.38 and 3.74, respectively. Moreover, the analysis has shown that our proposed method has a high level of reliability in predicting impulsive wave velocity using a neural network.

## Introduction

Numerous applications, such as surface nano structuring, laser micromachining, laser cleaning, laser shock processing, pulsed laser deposition, and nanofabrication, need an understanding of the characteristics in a laser ablation plume. Among the methods used to detect pressure during laser-matter interaction are hydrophones, piezoelectric transducers, fast photography, interferometry, probe beam deflection, and photoacoustic probe beam deflection using a broadband piezoelectric transducer. Optical thermometry, spectrally resolved pyrometry, and spectroscopy are a few methods for measuring temperature. The earliest studies on laser-induced shockwaves were published in the late 1960s ^1^. Measuring the velocity of shock waves as a function of time, for example via shadowgraph imaging, is a common method for studying shock waves generated by lasers ^2^. It is thus essential to comprehend how to accurately measure the pressure features of shock waves generated by lasers and how to build a connection between laser parameters and shock wave pressure characteristics ^3^. Since it is the most direct component influencing the target's response state, this research of the properties of laser-induced shock waves is significant. The purpose of this study is to provide a learning model that can minimize error and properly forecast shock wave velocity. Using an optimization technique makes our work method creative. Using this method, we try to not only train the neural network but also alter the design of the network. We will also be able to determine the optimal architecture for the network to enhance prediction accuracy. Our main contributions are summarized as follows in brief:

- Modelling the pattern of shock wave speed changes based on time with an artificial neural network.
- Applying the genetic algorithm to adjust the neural network's weight vector.
- Accurate measurement of shock wave velocity based on different configurations.

The following is the paper's continuation: In the next section, we examined various related studies. The materials and technique were presented in the third section. The findings are evaluated in the fourth section, and the fifth and final section closes up.

## Related works

A qualitative relationship between shock-wave velocity and pressure was found by Radziejewska et al. ^4^ when they examined the velocity and pressure of a shock wave produced by a nanosecond pulse laser with a wavelength of 1064 nm and a pulse width of 12 ns. The greater the shock-wave velocity, the greater the pressure. Zhu et al. ^5^ measured shock wave pressure profiles in copper and aluminum through PVDF using a 3 mm thick Poly-Methyl Methacrylate (PMMA) solid as a confining layer. When PMMA solids were utilized as the confining layer instead of direct irradiation, the shock wave pressure increased by a factor of 7.5–11.8.

Similar studies by Sarno et al. ^6^ examined the pressure of plasma shock waves in both air and water. According to studies, plasma expands in water at the speed of sound around 20 times more quickly than it does in air. A picosecond pulse laser with a 532 nm wavelength and a 10 ps pulse width was used by Daniel et al. ^7^ to irradiate copper. However, the mass specific thrust of a triple pulse laser is about the same as that of a single pulse laser. This is mainly because the ablation effect of the third pulse laser is much greater than that of the double pulse laser. Using two-dimensional emission and shadow imaging methods, Kiran et al. ^8^ examined the interaction between two plasmas and shock waves created by 532 nm nanosecond laser pulses with a pulse width of 7 ns. The shock wave and plasma development were depicted as functions of the distance and energy ratio between the two plasma sources. It was found that a plasma jet was formed when the shock wave from the high-energy plasma source passed through the lower-energy plasma. As the energy ratio increases, the diameter of the plasma jet will increase.

Mayer et al. ^9^ created a novel constitutive model to characterize high strain rates and severe stresses by using machine learning and molecular dynamics data. To capture the dynamic process of plasticity and approximate state functions, they used artificial neural networks. The axisymmetric deformation of a single crystal of copper was effectively investigated using the model; in particular, the loading resulting from plane shock waves was addressed. The dynamics of spherical laser-induced cavitation bubbles in water were investigated by Liang et al. ^10^ utilizing probe beam scattering, time-resolved shadowgraphs, and plasma photography. According to the study, the bubbles' gas concentration may be inferred from the frequency of their late oscillations. According to this research, the collapsed bubble had a pressure of 13.5 GPa and included more vapor than gas. Byun et al. ^11^ created a two-component seedless velocimetry technique that makes advantage of shockwaves produced in laser-induced plasmas (LIP) by concentrated nanosecond laser pulses. Two pairs of continuous-wave laser beams, each produced by a diode laser, are used in this procedure. Four probe beams in an air jet are used to evaluate the velocimetry approach, and an empirical point-explosion blast model is used to follow the shockwave's propagation. With the use of hot wire, the speed accuracy is confirmed to be 3 m/s. The velocity components are measured with an uncertainty of 4–6 m/s. The spatiotemporal evolution of shock waves produced by Nd:YAG lasers on copper targets at different pump laser power densities under atmospheric pressure circumstances was investigated by Alnama et al. ^12^. The scientists identified a supersonic shock wave that propagated and subsequently degraded into an acoustic wave using optical beam deflection. The results show that the temporal development of the shock front cannot be effectively predicted by drag force or point-explosion blast wave models.

The first dynamics of a shock wave created in stagnant water by a nanosecond pulsed laser were studied by Lai et al. ^13^. The technique examined the shock wave's quick temporal and spatial development and forecasted notable attenuation features. Using a two-dimensional numerical model, Wang et al. ^14^ examined the behavior of the pulsed laser-induced shock wave (PLISW) in laser micro- and nanostructure processing. Their investigation showed that the PLISW's development was influenced by a number of laser factors, including energy density, spot size, and duration. It was found that after processing with a nanosecond laser, the overpressure and wavefront velocity increased quickly. Peak overpressure values for energy densities of 3.4 J/cm^2^ and 4.3 J/cm^2^ were found to be 110 MPa and 167 MPa, respectively. Furthermore, the shock wave exhibited irregular properties in both axial and radial directions as the laser spot size grew. Classification and Regression Tree (CART) analysis was first presented by Lewis et al. ^15^ as a machine learning method for classification and regression applications. In order to create decision trees for classification and regression, the input space is divided into binary tree structures. While CART is simple to understand, if it is not properly regularized, it may produce overfitted models.

Jang ^16^ the recently introduced adaptive-network-based fuzzy inference system (ANFIS) is a hybrid computational model that models complicated nonlinear connections by combining neural networks with fuzzy logic. It is used in data mining, pattern identification, time series prediction, control systems, and forward and backward passes using gradient descent. The impact of a combined pulse laser (CPL) with millisecond (ms) and nanosecond (ns) pulses on the shock-wave velocity of silicon was investigated by Li et al. ^17^. To forecast this velocity, they used a convolutional neural network (CNN) using input parameters including time, pulse delay, ms and ns laser energy density. The behavior of Zr-1Nb alloy under laser-induced shock wave loading was studied by Ledon et al. ^18^, with a particular emphasis on the material's behavior in the ultrafine-grained state. This alloy has garnered attention for improving the dependability of fuel rods and is often used in the manufacture of nuclear reactor fuel shells. According to this research, Zr-1Nb showed greater dynamic elastic limit and spall strength when it was in a coarse-grained condition. However, in an ultrafine-grained condition, Zr-1Nb showed a higher vulnerability to spall fracture.

Arai et al. ^19^ evaluated the hardness of the flesh of a soft mango fruit by inducing a Rayleigh wave using a laser-induced plasma shock wave method. We measured and looked for changes in firmness due to ripening in the Rayleigh wave propagation velocity, which is related to density, firmness, and Poisson's ratio. After 96 h of storage, the results showed a drop of almost 4% in the Rayleigh wave propagation velocity. Horvat et al.'s study ^20^ mimicking the anterior portion of the human eye, looked at the formation of secondary cavitation in aqueous medium after laser-induced breakdown. For different sites of the shock wave source, the first cavitation location was predicted using a numerical simulation. The study demonstrated large, brief negative pressures near the shock wave source's acoustic image, which caused a great deal of secondary cavitation.

In the area of laser-material interaction, researchers are committed to finding solutions for nonlinear issues. Predicting the characteristics of the target material after laser shock peening is their primary goal ^21,22^. As an example, Wu et al. ^23^ use an artificial neural network to forecast the mechanical properties of titanium alloy after laser shock peening. The test data set's correlation index R2 is between 0.997 and 0.987, based on the findings. The least squares method was used, for example, by Matsui et al. ^24^ to forecast the detonation wave velocity supported by a laser. With an increase in laser energy density, the velocity of the detonation wave supported by the laser seems to rise.

## Research methodology

The present research was conducted in two phases, laboratory experiments and simulations. In the laboratory phase, the shock wave velocity was precisely measured based on different configurations. The data collected in the laboratory phase were then used in the second phase to train an artificial neural network model to learn the pattern of shock wave velocity.

### Measurement of shock wave velocity

**Figure 1:**
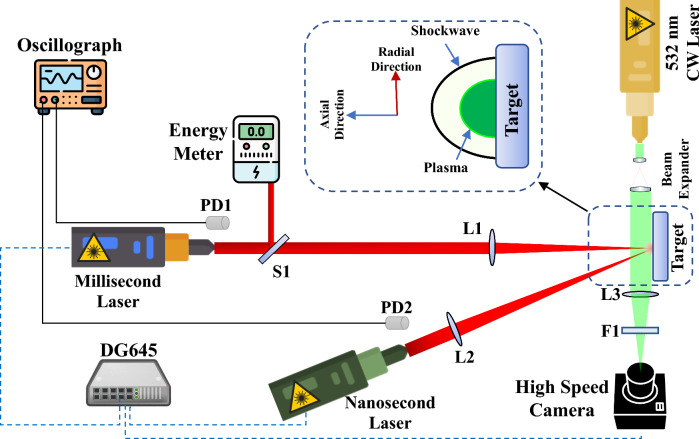
Experimental setup configuration for shock wave velocity.

Using a combined pulse laser system, experiments on laser-induced shock waves were conducted in accordance with the design described in ^17^. The pulse lengths of the millisecond and nanosecond lasers were set to 1 ms and 10 ns, respectively, while the laser wavelength was set to 1064 nm. Furthermore, the repetition rate was adjusted to 1 Hz, and the target diameters for these two lasers were set to 1 and 1.3 mm. Figure 1 shows the layout of the experimental setup.

Quantification The beams of nanosecond and millisecond lasers converge at a same target point at a 5-degree angle after passing through lenses with a 50-cm focal length. As in ^17^, the shadowgraphy approach is used to track the propagation of shock waves. Through a beam expander, a continuous wave laser operating at 532 nm is used as a background light source to impinge perpendicularly on the area in front of the target where shock waves are formed. An energy meter and spectrometer are used to determine the millisecond laser's energy. An oscilloscope and two optical detectors that correspond to each laser are used in the experiments to measure the delay of the two millisecond and nanosecond laser pulses.

The delay is controlled by a digital delay/pulse generator, DG645. The following formula may be used to determine the shock wave velocity:

1 $${V}_{sw}\left(k\right)=\frac{p\left(k+{d}_{k}\right)-p(k)}{{d}_{k}}$$

The formula above denotes the time interval between two observations as ${d}_{k}$, and the greatest radial propagation distance of the shock wave at time step k as $p(k)$ which is in units of length and defined in centimeters.

### Artificial neural network-based prediction of shock wave velocity

In the second phase of the research, an artificial neural network is used to model the pattern of shock wave velocity changes over time. To do this, 9 experiments with different configurations are initially conducted, and the data collected from these experiments are organized as structured data records. During each experiment, the shock wave velocity is measured at time intervals $t=\{\text{7,12,17,22,27,32}\}$ microseconds, and these values are considered as the target variable (dependent variable) in the estimation problem. By following this process, each data record is described by 5 features:

- Nanosecond Laser Energy Density (Df): This independent variable is described in joules per square centimeter and can take one of the values ${D}_{f}=\left\{\text{6,12,24}\right\}\frac{j}{c{m}^{2}}$ in different configurations. In configurations 1 to 3, this parameter is set to 6, in configurations 4 to 6, it's set to 12, and in other configurations, it's set to 24.
- Millisecond Laser Energy Density (Dm): The unit of measurement for this independent variable is also joules per square centimeter and can have one of the values ${D}_{m}=\left\{\text{0,226.3}\right\}\frac{j}{c{m}^{2}}$ in different experiment setups. In configurations 1, 4, and 7, the value of Dm is set to zero, and in other configurations, it's set to 226.3.
- Pulse Delay (Dt): The pulse delay of the millisecond and nanosecond lasers in the experiments is controlled through a digital delay/pulse generator DG645 and monitored via an oscilloscope and two optical detectors corresponding to each laser. In the conducted experiments, the pulse delay can take one of the values ${D}_{t}=\left\{\text{0,0.4,0.8}\right\}ms$ in different configurations. In configurations 1 to 3, this parameter is set to zero, in configurations 4 to 6, it's set to 0.4, and in other configurations, it's set to 0.8.
- Time (t): This independent variable represents the time intervals at which the shock wave velocity is measured and, for each configuration, includes all values $t=\left\{\text{7,12,17,22,27,32}\right\} \mu s$ microseconds.
- Shock Wave Velocity (${V}_{sw}$), which is considered as the dependent variable in this phase of the proposed method, aims to model the relationship between the independent variables ${D}_{f}, {D}_{m}, {D}_{t}$, and $t$ with ${V}_{sw}$ through an artificial neural network.

In this phase of the research, an attempt is made to predict the value of ${V}_{sw}$ using the variables ${D}_{f}, {D}_{m}, {D}_{t}$, and $t$. If we denote the relationship between the mentioned independent variables and ${V}_{sw}$ as $f$, then we will have:

2 $${V}_{sw}=f({D}_{f},{D}_{m},{D}_{t},t)$$

The current research aims to model the function f using artificial neural networks. While some recent studies, such as ^17^, have attempted to model this function using convolutional neural network (CNN) models, it should be noted that the problem of modeling f in this research has characteristics that are incompatible with CNN models. Firstly, the number of data records collected through experiments with different configurations may not meet the requirements of deep models like CNNs, which can increase the risk of overfitting. Secondly, the data structure in the current problem is not compatible with the processing objectives of CNN models, making it challenging to ensure the general applicability of deep models in real-world conditions. These characteristics led to the use of multilayer perceptron (MLP) networks for predicting shock wave velocity in the current research.

While MLP models can address data compatibility concerns compared to CNN models, optimizing the MLP model configuration is still necessary to achieve accurate predictions. Using a large number of neurons and layers in the MLP model can increase model complexity, potentially leading to reduced prediction accuracy. Additionally, conventional training algorithms for adjusting neural network weights may not guarantee the highest prediction accuracy.

To address these challenges, the proposed method uses genetic algorithms for optimizing the configuration of the MLP model and training it.

**Figure 2:**
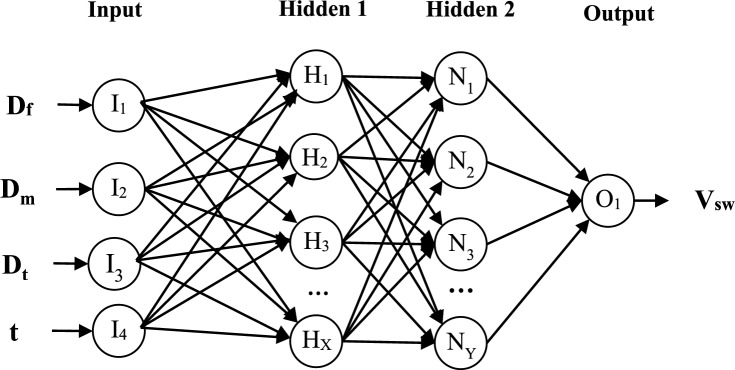
Neural network structure in the proposed method for predicting shock wave velocity.

The suggested technique modifies the neural network's weight vector by using genetic algorithms in place of conventional training techniques. Apart from setting the hidden layers of the MLP model, this optimization model looks for the best weight vector for the neural network by using the training error criteria (as its fitness function). Figure 2 shows the neural network architecture used in the suggested technique for estimating shock wave velocity.

As a result, the suggested MLP's design is dynamic. These two hidden layers have logistic sigmoid and linear activation functions, respectively. Lastly, the output layer has a single neuron that reflects the input sample's expected shock wave velocity. In Fig. 2, the weighted inputs of each neuron in this neural network are shown as vector arrows. To keep things simple, each neuron also has a bias value, but this isn't included in the figure specifically. In this configuration, the output of each neuron for transmission to the next layer is calculated as follows:

3 $${y}_{i}=G(\sum_{n=1}^{{N}_{i}}{w}_{n}\times {x}_{n}+{b}_{i})$$

The input value is represented by ${x}_{n}$ in the equation above, the weight of the nth neuron is represented by ${w}_{n}$, and the bias of that neuron is described by ${b}_{i}$. In addition, G(.) explains the activation function and ${N}_{i}$ denotes the number of inputs for neuron i. The response vector's structure and the fitness assessment standards are discussed in the parts that follow. After that, the procedures for setting up and training the MLP model using a genetic algorithm are covered. The architecture of the MLP, the weights of the connections between neurons, and their biases are all determined by the response vector (chromosome) used in the evolutionary algorithm in the suggested technique. As a result, the optimization algorithm's response vectors are made up of two connected parts. As a result, the genetic algorithm's chromosome lengths are changeable and depend on the neural network's architecture. For each hidden layer in the network, a range of 0 to 15 neurons is taken into consideration, since there is an unlimited number of potential network topologies. As a result, every element in the response vector's initial section is a natural integer between 0 and 15, and if any of them are zero, the associated layer will be eliminated. It's important to note that only the number of neurons in hidden layers is defined in the first section of the chromosome (the topology determination portion), as the network has a single output neuron and the dimensions of the input layer are determined by the number of input characteristics. For a neural network with I input neurons, H1 neurons in the first hidden layer, H2 neurons in the second hidden layer, and P output neurons, the length of the second part of each chromosome in the genetic algorithm will be equal to:

4 $$L={H}_{1}\times \left(I+1\right)+{H}_{2}\times \left({H}_{1}+1\right)+P\times \left({H}_{2}+1\right)$$

**Figure 3:**
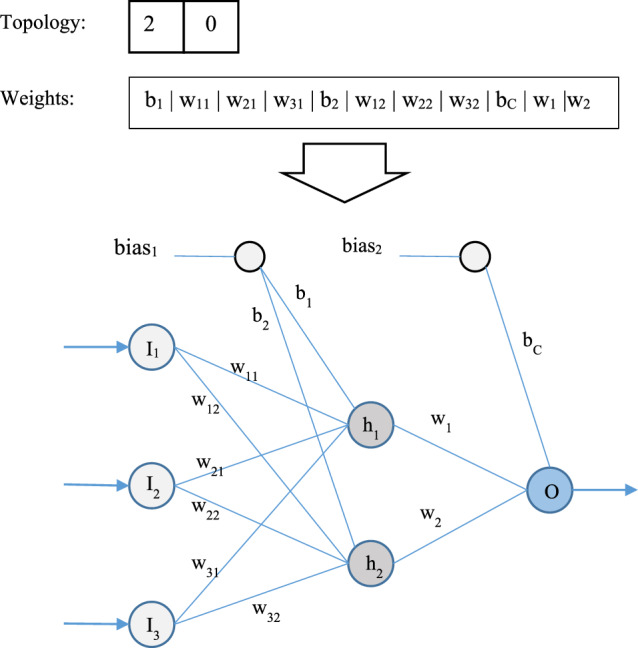
A sample response vector for determining the topology and weight vector of the neural network.

In the above equation, ${H}_{1}\times \left(I+1\right)$ represents the number of weight values between neurons of the input layer and the first hidden layer plus the bias of the first hidden layer. The value ${H}_{2}\times \left({H}_{1}+1\right)$ indicates the number of weights between the first and second hidden layers plus the bias of the second hidden layer. Finally, $P\times \left({H}_{2}+1\right)$ represents the number of weights between the last two layers plus the bias of the output layer. In this vector, the weight and bias are described as real numbers in the range [− 1, + 1]. In other words, each optimization variable in the second part of the chromosome is described as a real variable with search boundaries of [− 1, + 1]. A sample response vector for a network is shown in Fig. 3.

Figure 3 defines the architecture of the network as a network with one hidden layer and two neurons in the first section of a hypothetical response vector. This means that the second half of the response vector for figuring out the weights of neurons and biases of the neural network will have a length of 11 if the number of input features is 3 (for simplicity) and the number of output neurons is 1. A simplified representation of the final neural network's topology may be seen at the bottom of Fig. 3. It is important to remember that the genetic algorithm randomly selects the starting population. The neural network outputs are created for training samples and compared to the real goal values (ultrasonic wave velocity) after the weights are determined using this response vector. As a result, the genetic algorithm's fitness function will be defined as follows:

5 $$MAE=\frac{1}{N}\sum_{i=1}^{N}|{T}_{i}-{Z}_{i}|$$

Also, ${Z}_{i}$ denotes the output produced by the neural network for the i-th training sample. Due to the encoding of chromosomes and the evaluation function for their fitness, the configuration and training steps of the MLP model using the genetic algorithm will be as follows:

Step 1: An initial population of N chromosomes is randomly created.

Step 2: The fitness of each chromosome is calculated based on Eq. (5).

Step 3: Using the roulette wheel algorithm, 0.8×N parent chromosomes are selected.

Step 4: Parent chromosomes are combined through two-point crossover to create child chromosomes.

Step 5: Each child chromosome has a mutation probability of ${N}_{m}$. During the mutation process, m random positions (from the second part of the chromosome) are selected, and these positions are randomly replaced with values within the search bounds.

Step 6: The fitness of the generated child chromosomes in the current population is calculated using Eq. (5).

Step 7: The child chromosomes created in the current population are merged with the previous population, and the resulting set is sorted in ascending order based on fitness. Then, N top chromosomes in this set are chosen as the new generation population.

Step 8: The chromosome with the lowest fitness in the new generation is retained as the best-found solution.

Step 9: If one of the following termination conditions is met, Step 10 is executed, and in this case, the algorithm repeats from Step 3. The termination conditions of the genetic algorithm in the proposed method are as follows:

- If the minimum fitness value does not change for a certain number of consecutive generations, ${N}_{t}=100$.
- If the number of algorithm iterations reaches a predetermined fixed value, G = 800.

Step 10: The configuration and weight vector associated with the chromosome with the best fitness are returned as the optimal solution.

## Research findings

The proposed approach was simulated using MATLAB 2020a. We employed a 6-repetition cross-validation technique to conduct the studies. Our proposed method has been implemented in three modes: the proposed method, Levenberg–Marquardt (trainLM), and scaled conjugate gradient backpropagation (TrainSCG). In the proposed method, neural network training and optimization is done using genetic algorithm. In ANN (trainLM) and ANN (TrainSCG) methods, trainLM and TrainSCG algorithms are used to train neural networks, respectively. The aim of the proposed method, compared to this approach, is to determine whether optimizing the topology and training of the network through a genetic algorithm can have a positive impact compared to conventional training algorithms or not. We have also compared the proposed methods with three papers: CART ^15^, ANFIS ^16^, and CNN ^17^.

**Figure 4:**
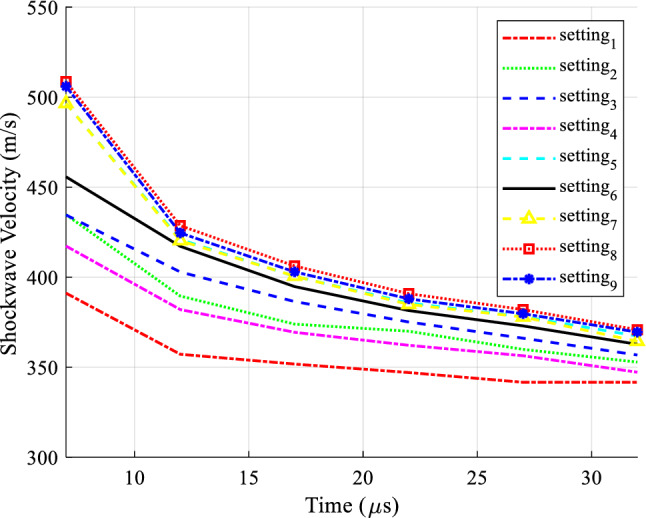
Information on nine time settings.

Figure 4 shows the information related to different settings for shock wave speed in terms of time in 9 different modes, this figure shows the actual speed values calculated for different samples.

**Figure 5:**
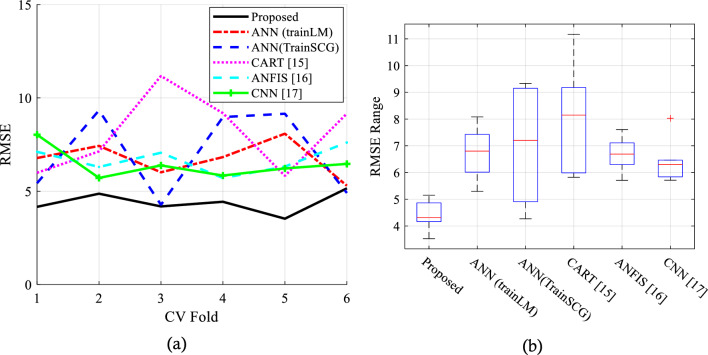
Evaluation of the RMSE of the predicted target.

Figures 5a,b show the RMSE values and the average error values in different iterations. Based on Fig. 5, the goal is to achieve a lower prediction error for this metric. This is supported by analyzing the RMSE variations and studying the boxplots. The proposed method consistently exhibits lower squared error and higher accuracy with lower RMSE in comparison to other scenarios. The lower level of prediction error observed in Fig. 5a and the narrower range of error variations shown in Fig. 5b indicate a higher probability of the proposed method's outputs being correct, as compared to the other scenarios. The RMSE measure is calculated in these graphs using the following equation:

6 $$RMSE=\sqrt{\frac{1}{N}} {\sum }_{i=1}^{N}{\left({y}_{i}-{\widehat{y}}_{l}\right)}^{2}$$

**Figure 6:**
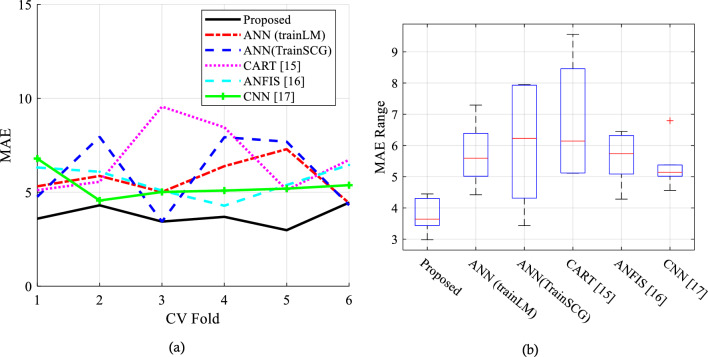
Evaluation of the MAE of the predicted target.

Figure 6a shows the MAE values at each iteration, demonstrating that the proposed method achieves lower MAE in predicting the target variable. The figure also highlights that the proposed method exhibits a narrower range of absolute error variations across different iterations, which enhances its reliability as an algorithm. In Fig. 6b, the box plot of MAE after 6 iterations is presented, where the solid line within each box represents the upper and lower bounds of the absolute error variations observed throughout different iterations. When compared to other methods, the proposed method showcases a lower MAE and a narrower range of absolute error variations. Moreover, the proposed method demonstrates higher average accuracy than other methods. The narrower range of absolute error variations in the proposed method indicates a higher level of reliability. The proposed method has an error rate of 3 to 4 and a half meters per second, which is significantly lower than prior methods. Furthermore, the proposed method's maximum error value is at least 50% lower than the compared approaches. To sum up, in comparison to other methods, the suggested method is more precise and dependable in predicting the target variable. The proposed method's reliability is further strengthened by its smaller range of absolute error variations.

**Figure 7:**
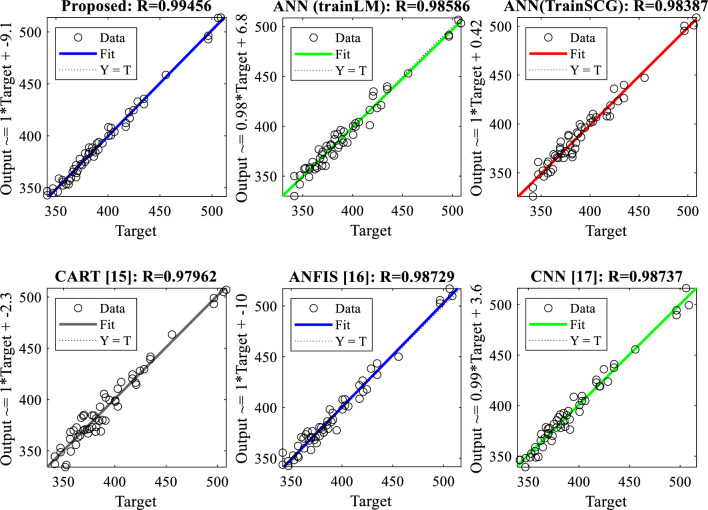
Liner regression plot for the shock wave velocity prediction.

The regression plot in Fig. 7 shows the relationship between each model's projected values and actual values. Our suggested approach has the greatest prediction score of 0.99456 out of all the strategies assessed. The ANFIS approach ^16^ has a score of 0.98729, which is closely followed by the CNN method ^17^ with the second closest prediction score of 0.98737. This clearly shows how much better our method is.

**Figure 8:**
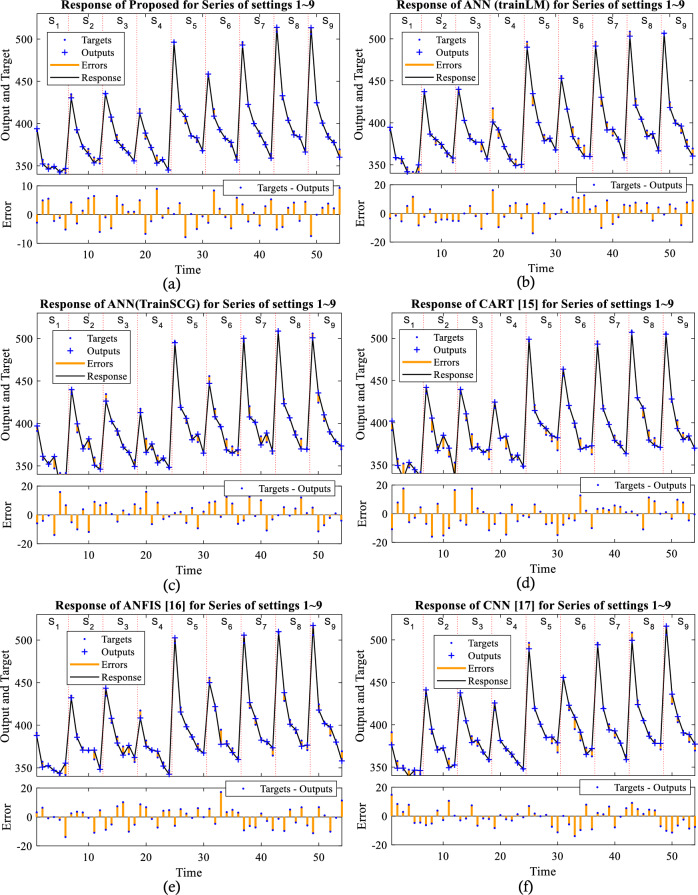
Response plot for the shock wave velocity prediction.

The black line corresponds to the response chart, and the outputs of the proposed method are displayed as character + while the actual values are shown as dots. The difference between them, represented by an orange line connecting the values, indicates the error. Thus, we can observe the magnitude of error variations for different samples in different settings. In our proposed method, the error is higher in settings 2 to 5 compared to other methods, which can be attributed to changes in range and the degree of anomaly in the data associated with these settings. In the lower section of the chart, it also represents the error values on the horizontal axis. Here, it can be observed that the error rate for the proposed method is close to zero, while our method has a lower error rate. The worst-case scenario for the error can range between + 10 and − 10 (see Fig. 8).

**Figure 9:**
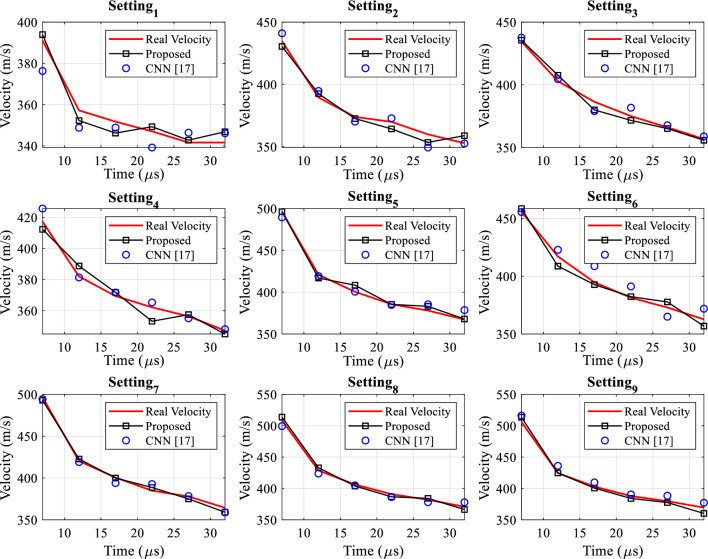
Comparison of shock wave velocity prediction with the proposed method and the CNN model ^17^.

Figure 9 compares the predictions of our proposed method with the closest CNN model ^17^ under nine different settings. The speed values are plotted over time, with the real scenario indicated by the red line, the predicted results of our proposed method represented by the black line, and the circular markers denoting the CNN ^17^ paper. In all settings, our proposed method has been able to provide more accurate estimations and reduce the error compared to the comparative approach in all settings. The utilization of a dynamic structure for the neural network and its dynamic configuration through optimization algorithms can be effective in improving prediction performance.

**Figure 10:**
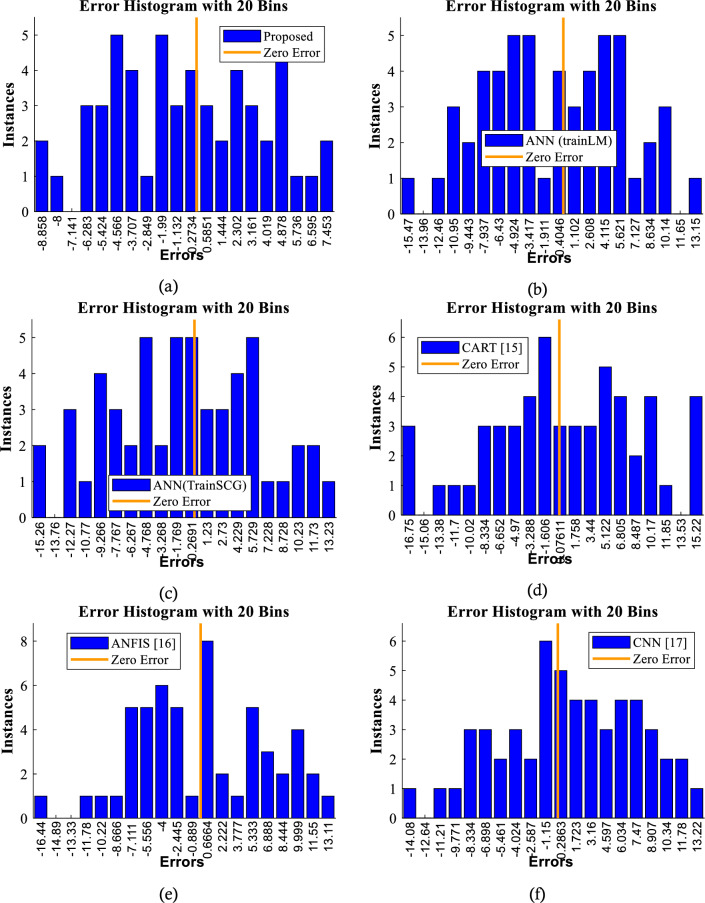
Evaluation of error histograms.

The performance of the prediction algorithm can be fully understood by looking at the error histogram intervals. Figure 10 depicts the error histograms for various ways of forecasting shock wave velocity. The vertical axis displays the frequency of predictions falling within each interval, while the horizontal axis depicts the error intervals. Plots show that, in comparison to the other ways, our suggested solution has a smaller error interval. This suggests that our approach predicts shock wave velocity more correctly. Our suggested method's error boundaries are [− 8.8, + 7.4]. This basically indicates that our approach predicts the target variable with a maximum error of 7.4 grades, even in the worst-case situation. This inaccuracy is insignificant when compared to the errors of other instances.

Performance indicators including mean absolute error (MAE), concordance correlation coefficient (CCC), and root mean square error (RMSE) are included in the figures under each approach. It is clear that the suggested approach performs better since it has the lowest RMSE and MAE values as well as the highest CCC value. However, the CNN technique also yields almost-ideal outcomes in terms of performance measurements. The CCC measure is calculated using the following equation:

7 $$CCC\left(T,P\right)=\frac{2\times Corr(T,P)\times {\sigma }_{T}\times {\sigma }_{P}}{{\sigma }_{T}^{2}+{\sigma }_{P}^{2}+{\left({\mu }_{T}-{\mu }_{P}\right)}^{2}}$$

where T and P represent the target and output vectors, respectively. Also, $\sigma$ and $\mu$ demonstrate the standard deviation and mean values of the vector. Finally, $Corr(T,P)$ demonstrate the Pearson correlation coefficient between target and output vectors (see Table 1).

**Table 1:** The efficiency of the proposed method in comparison to alternative approaches.

Method	RMSE	MAE	CCC
Proposed	4.3883	3.7426	0.9941
ANN(trainLM)	6.7375	5.7176	0.9855
ANN(TrainSCG)	7.0112	6.0131	0.9835
CART ^15^	8.0768	6.7551	0.9791
ANFIS ^16^	6.6847	5.6030	0.9865
CNN ^17^	6.4410	5.3376	0.9870

## Conclusion

Precise estimation of shock wave speed is essential for understanding its behavior and using it in disciplines such as material science, aeronautical engineering, and medicine. Neural networks provide a potential alternative to traditional approaches, which often depend on empirical calculations. They are capable of learning from data and recognizing intricate patterns. However, by employing neural networks, which are capable of learning complex patterns from data, the accuracy of shock wave velocity prediction can be greatly enhanced. The dynamic design of the neural network structure, coupled with the optimization capabilities of genetic algorithms, allows for fine-tuning the network's weights and determining its topology, resulting in more precise predictions. The results demonstrate that our recommended method, which uses the configuration modification strategy and genetic algorithm training, has been able to reduce the MAE value by at least 3.74 and the RMSE value by 4.38 when compared to conventional training methods like ANN (trainLM) and ANN (TrainSCG). These findings show that the neural network-based technique shows tremendous potential for increasing the forecast accuracy of laser-induced shock wave velocity, as it considerably outperforms other comparing approaches.

## Data Availability

All data generated or analysed during this study are included in this published article.
